# Use of Self-Complementary Adeno-Associated Virus Serotype 2 as a Tracer for Labeling Axons: Implications for Axon Regeneration

**DOI:** 10.1371/journal.pone.0087447

**Published:** 2014-02-03

**Authors:** Yingpeng Liu, Kathy Keefe, Xiaoqing Tang, Shen Lin, George M. Smith

**Affiliations:** Shriners Hospitals Pediatric Research Center, Temple University School of Medicine, Philadelphia, Pennsylvania, United States of America; Hertie Institute for Clinical Brain Research, University of Tuebingen., Germany

## Abstract

Various types of tracers are available for use in axon regeneration, but they require an extra operational tracer injection, time-consuming immunohistochemical analysis and cause non-specific labeling. Considerable efforts over the past years have explored other methodologies, especially the use of viral vectors, to investigate axon regeneration after injury. Recent studies have demonstrated that self-complementary Adeno-Associated Virus (scAAV) induced a high transduction efficiency and faster expression of transgenes. Here, we describe for the first time the use of scAAV2-GFP to label long-projection axons in the corticospinal tract (CST), rubrospinal tract (RST) and the central axons of dorsal root ganglion (DRG) in the normal and lesioned animal models. We found that scAAV2-GFP could efficiently transduce neurons in the sensorimotor cortex, red nucleus and DRG. Strong GFP expression could be transported anterogradely along the axon to label the numerous axon fibers from CST, RST and central axons of DRG separately. Comparison of the scAAV2 vector with single-stranded (ss) AAV2 vector in co-labeled sections showed that the scAAV2 vector induced a faster and stronger transgene expression than the ssAAV2 vector in DRG neurons and their axons. In both spinal cord lesion and dorsal root crush injury models, scAAV-GFP could efficiently label the lesioned and regenerated axons around the lesion cavity and the dorsal root entry zone (DREZ) respectively. Further, scAAV2-GFP vector could be combined with traditional tracer to specifically label sensory and motor axons after spinal cord lesion. Thus, we show that using scAAV2-GFP as a tracer is a more effective and efficient way to study axon regeneration following injury.

## Introduction

Axonal tracing using tracer molecules such as BDA, CTB or fluorogold has been an essential tool for many years in studying axonal structure and continuity [1], [2]. However, this approach has certain drawbacks. It requires time consuming procedures such as tracer injections and immunohistochemical staining, can cause non-specific labeling, and limits detection to a specific time window [3]. Previous studies showed that AAV2 vectors provide distinct advantages over classic anterograde tracers and these vectors have the ability to transduce neurons efficiently and transport to axon terminals, which allow entire axons to be labeled [4], [5], [6], [7]. Neuronal transduction by conventional AAV vectors has been demonstrated in the cortex, brainstem, and sensory ganglia [5], [4], [8]. However, conventional AAV vectors carry a single-stranded (ss) DNA genome, which must be converted by host-cell-mediated DNA synthesis to double-stranded DNA for transgene expression. This requirement for formation of double-stranded DNA has proven to be an important limiting factor for AAV vector transduction. The recently developed self-complementary AAV (scAAV) vectors provide an invaluable tool in AAV-mediated gene therapy studies for CNS diseases due to their faster expression and high transduction efficiency [9]. The application of scAAV vectors in normal and lesioned axonal labeling in the CST, RST and central axons of the DRG has not been fully investigated. In this study, scAAV serotype 2 vector was used to transfect neurons in the sensorimotor cortex, red nucleus and DRG to anterogradely trace their axons in normal and lesioned animal models. Our aim in this study was to determine whether scAAV2-GFP could serve as a suitable tracer to label long axonal tracts in animal models of CNS or PNS injury. This tracer can also be used in conjunction with other tracers to label multiple axon tracts.

## Materials and Methods

### Vector Construction and Production

Serotype 2 AAV vector was generated by helper virus-free transfection of 293 T cells with three-plasmid helper-virus free system. 293 T cells from the American Type Culture Collection (ATCC) were grown to 70–80% confluence at which point they were transfected with two packaging plasmids using PEI (Polyethylenimine, linear, MW-25 k, Warrington, PA): one carrying the AAV rep, cap genes and another helper plasmid carrying the adenovirus helper functions. The ssAAV and scAAV were type 2 vectors carrying the enhanced GFP (eGFP) or mCherry gene driven by the chicken beta actin promoter. The self-complementary GFP vector contains both the positive and negative strand of the DNA to be packaged in the vector. Three days after transfection, cell lysates and supernatants were harvested and 40% PEG 8000 was added to precipitate crude virus for 2 hours. Adeno-associated virus from this preparation was purified by double-centrifugation with cesium chloride (CsCl) and the isolated virus was dialyzed in 0.1 M PBS/5% sorbital overnight [10]. The titers of scAAV-GFP and ssAAV-mCherry used in this study were determined by infecting fibroblast cells. Serial dilutions of scAAV2-GFP and ssAAV2-mCherry vectors were added to a 24-well plate, which had been seeded with 0.5×10^5^ fibroblast cells per well. After one day, fibroblast cells expressing GFP or mCherry were counted under the fluorescence microscope to determine the vector titer per ml and then converted to genomic copy titer/mL (GC/mL) [10]. The scAAV2-GFP was found to be 1.6×10^12^ GC/mL and the ssAAV2-mCherry was prepared to the same titer.

### Animals and Surgical Procedures

A total of twenty-five adult Fischer 344 rats (Harlan) weighing 175–200 g were used in this study. All surgical interventions and postoperative animal care were provided in accordance with the guide for the care and use of laboratory animals, and the guidelines for rodent survival surgery provided and approved by the Institutional Animal Care and Use Committees of Temple University. The rats were anesthetized by intraperitoneal injection of ketamine (67 mg/kg) and xylazine (6.7 mg/kg). To determine whether scAAV2-GFP can label the CST, RST and central DRG axons in an anterograde manner, scAAV-GFP was directly injected into the sensorimotor cortex, red nucleus or dorsal root ganglions, respectively. For the cortex and red nucleus viral injections, the animals were placed into a stereotaxic frame (David Kopf Instruments, Tujunga, CA). After exposure of the skull surface, a burr hole was made in the skull to expose either the sensorimotor cortex or the right red nucleus with a dental drill at the coordinates described previously (Sensorimotor cortex: anteroposterior (AP): 2.0 mm; Lateral (L): 2.0; dorsoventral (DV):1.8 mm; AP: 0 mm; L: 2.0 mm; DV: 1.8 mm; AP: −2.0 mm; L: 2.0 mm; DV: 1.8 mm. Red nucleus: AP: −6.1 mm; L: 0.6 mm; DV: 7.2 mm and AP: −5.88 mm; L: 0.6 mm; DV: 7.2 mm). For DRG AAV injection, the dorsal aspects of cervical levels 5, 6 and 7 spinal cord segments were exposed by performing a cervical laminectomy and associated DRGs were exposed. One microliter of viral vector was injected into each site of the cortex, red nucleus or DRG. Red nucleus injections were performed with a 30 G steel needle that was attached to a 10µl Hamilton syringe (Hamilton, Reno, NV). After the needle was lowered to the appropriate depth, AAV vectors were infused at a rate of 0.40 µl/min. After completion of the injection, the needle was left in place for 5 min to allow for the vector to diffuse away from the injection site. The cortex and DRG injections were performed using a nanoliter injection system (Nanoliter 2000, World Precision Instruments) mounted on a micromanipulator. Beveled tips with diameters of 20 to 40 µm were used for this purpose. AAV vectors were injected at a rate of 0.10 µl/min using a Micro4 microsyringe pump controller (World Precision Instruments) and the pipette was left in place for 5 min before removal to insure good viral diffusion into the tissue.

**Figure 1:**
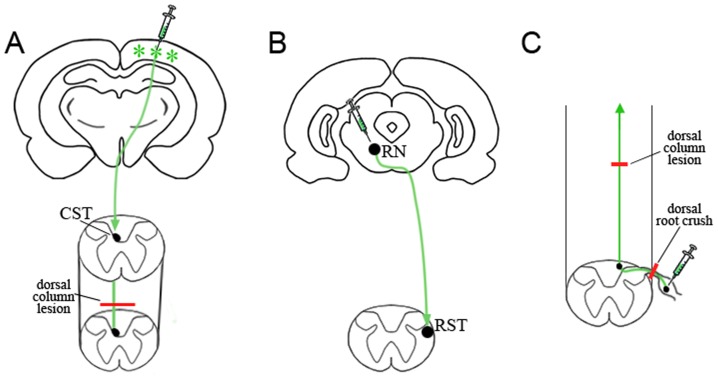
Schematic overview of experimental procedures. Schematic representations show pathways for the CST, RST and central axons of the DRG. A: The CST originates from the sensorimotor cortex, decussates at the medullary level of the brain stem and descends in the deep layer of the dorsal column. scAAV2-GFP vector was injected into sensorimotor cortex (green stars indicate GFP expression). Dorsal column lesion (red line in A) was performed to transect fibers of the CST in the cervical spinal cord. B: The RST runs from the neuronal bodies in the brainstem, crosses to the contralateral side and descends in the lateral white matter of the spinal cord. scAAV2-GFP was injected into the red nucleus (RN) to label the RST. C: Central axons of DRG originate from DRG neurons, pass through the dorsal root entry zone and ascend in the spinal cord dorsal column to reach the brain stem. Red lines in C indicate dorsal root crush and dorsal column lesions that transect the central axons of DRG in the dorsal root and spinal cord respectively. scAAV2-GFP was injected into DRG to anterogradely label central axons of the DRG.

For evaluation of scAAV2-GFP tracing of lesioned axons after injury, we performed dorsal column lesion and dorsal root crush injury on 12 animals. The animals receiving spinal cord dorsal column lesions underwent a laminectomy at C4 spinal level, and a Kopf microwire knife (Kopf Instruments, Tujunga, CA) was used to make the lesions at 1.5 mm-width and 1.5 mm depth from the dorsal spinal cord surface as previously described [11]. For dorsal root crush injury, the animals underwent a hemilaminectomy at the L1–L2 vertebral segments to expose the lumbar dorsal roots. With the use of #5 Dummond forceps, triple-crush lesions, 10 seconds each, were inflicted at two sites separated by 3 mm along the L4, L5 afferents. All lesions were performed unilaterally on the right side. For dorsal column lesions, scAAV2-GFP was injected into the sensorimotor cortex after injury to anterogradely trace the CST. In the model of dorsal root crush injury, scAAV2-GFP was injected into the DRGs at L4 and L5 after injury to anterogradely trace the central branch of dorsal root axons. To trace both sensory and CST axons in the dorsal column lesion model, we injected scAAV2-GFP into the cervical DRGs to label dorsal column sensory axons and performed dorsal column lesion with Kopf microwire knife (Kopf Instruments, Tujunga, CA). After one week we injected 10% BDA (Molecular Probes, Eugene, OR) into the sensorimotor cortex at the coordinates described above to label CST axons and waited for 2 weeks before euthanizing animals. After surgery, rats were placed on a temperature controlled heating pad to allow the animals to recover. (Figure 1 shows schematic overview of surgical methods).

### Tissue Processing and Immunohistochemistry

At the end of the 2–4 week survival period, the animals were sacrificed by injection of Fatal-Plus (Dearborn, MI) and perfused transcardially with 0.9% NaCl, followed by 4% paraformaldehyde (PFA) in 0.1 M phosphate buffer (PB, pH 7.4). The brains, spinal cords, dorsal roots and DRGs were removed, post-fixed in 4% PFA at 4°C overnight and moved to 30% sucrose in 0.1 M PB at 4°C for 2–3 days. Tissue blocks were embedded in M-1 Embedding Matrix (Kalamazoo, MI) and quick frozen on dry-ice. Brains were sectioned into six series at 30 µm coronally using a cryostat, and stained as free floating sections before being mounted onto slides. The spinal cords and DRGs were serially sectioned into five series at 16 µm using a cryostat and mounted directly on slides. To identify the nuclei of neuronal cells or neurons with axons, immunostainings of NeuN and Neurofilament were performed as previously reported [12], [13]. The sections were permeabilized and non-specific antigenic sites blocked with 0.3% Triton X-100/10% normal goat serum in 0.1 M PBS for 30 min at room temperature. Tissues were then incubated with rabbit-anti-NeuN antibody (1∶400, Millipore, Temecula, CA), mouse-anti- βIII tubulin (1∶1000, Promega) or rabbit-anti-GFAP antibody (1∶500, DAKO, Corporation) at 4°C overnight. In order to increase GFP signals, some sections from brain or spinal cord were stained with a mouse-anti-GFP antibody (1∶500, from Molecular Probes, Eugene, OR). In most cases, sections were viewed directly for GFP fluorescence.

For BDA reaction, sagittal sections were incubated with ABC reagent (Vector Laboratories) for 30 min and visualized with streptavidin 594 (1∶400, Invitrogen). The next day, sections were incubated with Texas red-anti-rabbit IgG, AMCA-anti-rabbit IgG and FITC-anti-mouse IgG (1∶400, Jackson ImmunoResearch, West Grove, PA) at room temperature for 60 min. After staining, sections were coverslip-mounted with Fluoromount-G (SouthernBiotech, San Diego, CA) and photographed under a Nikon microscope.

## Results

### Specific and Stable Neuronal Transduction

**Figure 2:**
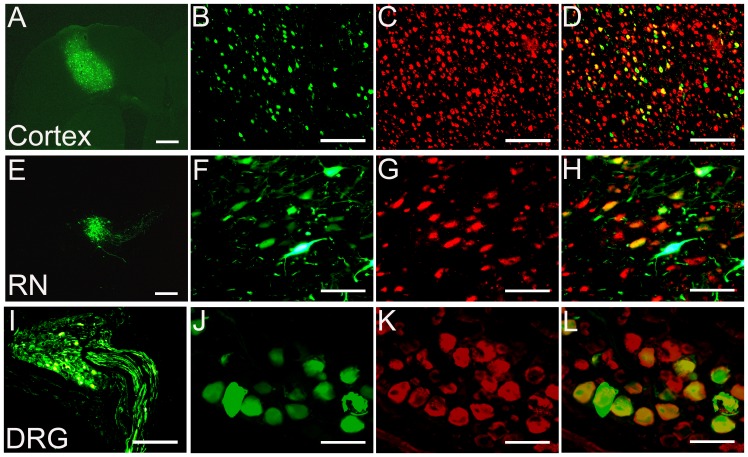
Specific neuronal transduction by recombinant scAAV2-GFP. Following injections of scAAV2-GFP into the sensorimotor cortex (A–D), red nucleus (RN) (E–H) and DRG (I–L), strong expression of GFP (green) was detected. The sections from these three areas were stained for NeuN (red) to specifically label the neurons (C, G and K). Scale Bars: 500 µm (A–E and I) and 200 µm (F–H, J–L).

The injection of scAAV2-GFP resulted in the transduction of numerous neuronal populations and GFP expression appeared to be greatly restricted to neurons in the sensorimotor cortex (Fig. 2A–D), red nucleus (Fig. 2E–H), or DRG (Fig. 2I–L), depending on injection site. To identify if GFP staining co-localized to neurons, Neuronal nuclear protein (NeuN) was used to specifically label neurons. Co-labeling was seen in many scAAV-GFP transduced cells in these three areas (Fig. 2D, H and L). Colabeling also indicated that scAAV-GFP transduced very few non-neuronal cells (data not shown). In order to examine the stability of GFP expression at different time points we compared the GFP expression level 2 weeks and 8 weeks following AAV injection. We found that the numbers of GFP-positive neurons at both time points did not change, demonstrating that GFP expression level was stable over time (data not shown). This neuronal selectivity of scAAV2-GFP demonstrates its use as a powerful tool for targeting neuronal populations for axon regeneration.

### Expression of AAV-scGFP in the Sensorimotor Cortex and CST Fibers in the Spinal Cord

**Figure 3:**
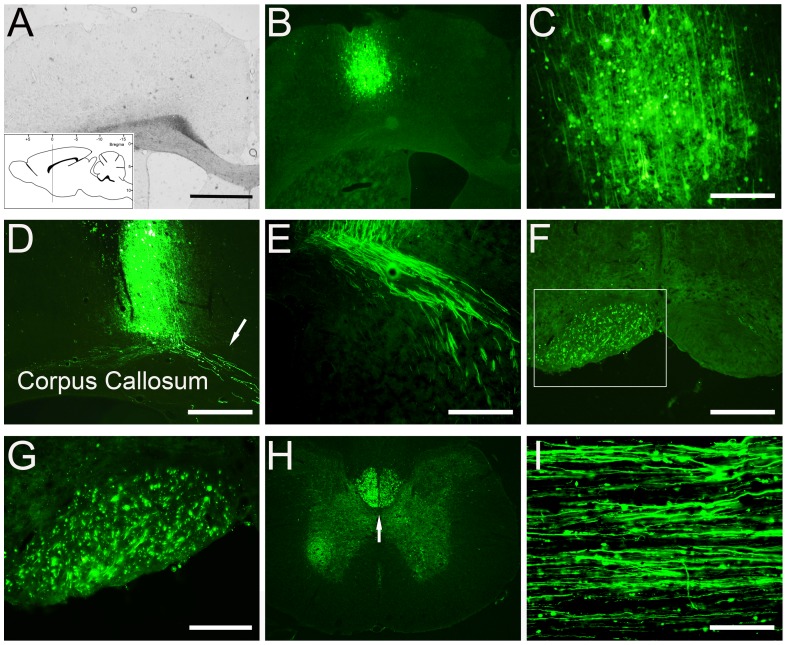
GFP expression in the sensorimotor cortex and anterograde labeling of CST axons. A shows the bright field of low-power image of sensorimotor cortex and corresponding low-power and high-power fluorescence images demonstrate high transduction efficiency of GFP (B and C). The schematic inset in panel A illustrates the coordinates and levels of this section**.** GFP positive CST fibers were found 4 weeks after viral injections above the corpus callosum (D and E), in the medulla (F and G), in the cervical spinal cord, in coronal (H) and sagittal (I) sections. The frame in the panel F shows a low magnification of the pyramidal tract, box represent high magnification in panel G. Animals used for panels A–E and H–I received bilateral viral injection. Animal for panels F and G received unilateral viral injections. Scale Bars: 500 µm (A, B and H), 200 µm (C, D and F) and 100 µm (E, G and I).

Following delivery of scAAV2-GFP to the primary sensorimotor cortex for 4 weeks, GFP expression was detected in the brain and the spinal cord (Fig. 3). For the brain, coronal sections revealed neurons expressing GFP at the injection site (Fig. 3B and C). At high magnification, proximal dendrites and axonal collaterals could be easily identified (Fig. 3C). GFP-labeled cortical fibers were observed along the corpus callosum (Fig. 3D and E). Examination of midbrain (Fig. 3F and G, which received unilateral cortical injections) and cervical spinal cord (Fig. 3H and I, which received bilateral cortical injections) revealed GFP-labeled CST fibers descending within the CST pathway. In the cervical spinal cord, GFP-positive fibers were found at the base of the deep dorsal columns following bilateral cortex injections (Fig. 3H). In sagittal sections numerous GFP-labeled CST fibers were also detected and found parallel to the longitudinal axis of the spinal cord (Fig. 3I).

### Transduction of AAV in the Red Nucleus and RST Fiber Labeling in the Spinal Cord

**Figure 4:**
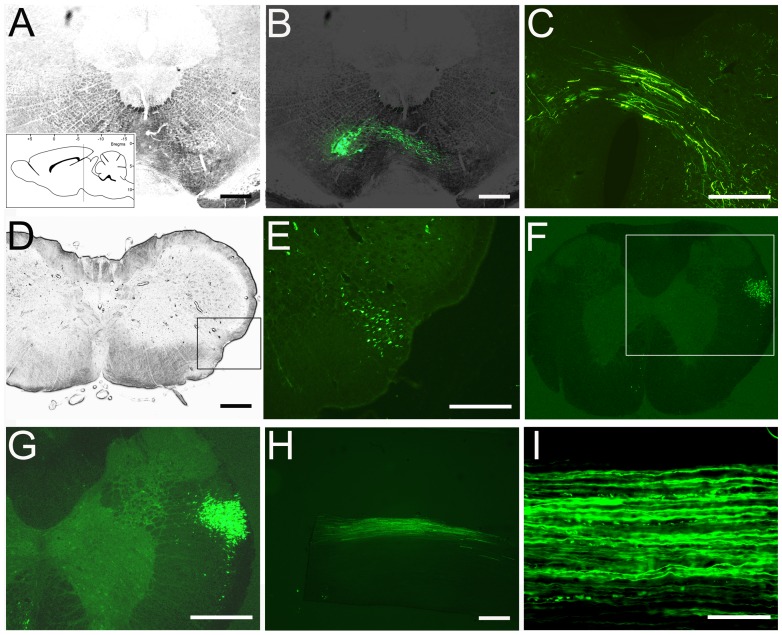
GFP expression in RN and anterograde labeling of RST. GFP expression was observed in the RN following scAAV2-GFP injection, as illustrated in coronal sections (A and B). The schematic inset in panel A illustrates the coordinates and levels of this section. Higher magnification images demonstrate anterograde transport of GFP to the contralateral site (C), midbrain (D and E) and cervical spinal cord in coronal (F and G) and sagittal sections (H and I). The box frames in panels D and F represent the regions magnified in panels E and G, respectively. Scale Bars: 500 µm (AH), 200 µm (I).

Stereotaxic scAAV2-GFP injections were performed into the right red nucleus and transduction efficiency of scAAV2-GFP in the red nucleus was examined at 2 weeks after AAV injections in coronal sections (Fig. 4). The scAAV2-GFP-transduced cells had a neuronal phenotype and high magnification imaging revealed transduced RN cells with long GFP-positive axons decussating to the contralateral site (Fig. 4B and C). GFP fluorescence was also found within the rubrospinal tract in the midbrain and cervical level 2 weeks after a unilateral scAAV2-GFP injection (Fig. 4E, F and H). In sagittal sections, GFP anterogradely transported to the cervical spinal cord showed straight GFP-labeled RST axons within the lateral funiculus (Fig.4H and I).

### Neuronal Transduction and Axonal Labeling After Direct Injection of scAAV2-GFP into the DRG

**Figure 5:**
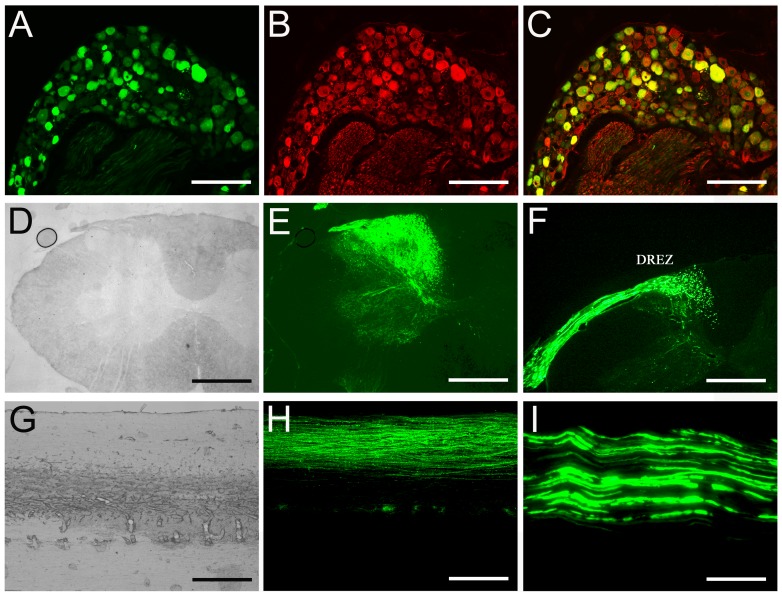
GFP expression in DRG and labeling of central and peripheral axons. GFP expressed in DRG neurons of the cervical spinal cord after scAAV2-GFP intraganglion injections (A–C). βIII-tubulin staining showed labeling in both neurons and axons (B and C). Many GFP positive fibers are visible in the dorsal horn (D and E), the dorsal roots (F), and can be traced over long distances to the cervical level of spinal cord (G and H). The peripheral nerve was also brightly labeled (I). Scale Bars: 200 µm (A–C, F and I), 500 µm (D–E, G–H).

GFP expression was examined after injecting scAAV2-GFP directly into C5–C7 DRGs. At 1 week post-injection, many neurons were transduced and expressed GFP (data not shown). Four weeks after AAV viral injections, DRG sections were stained with βIII tubulin antibody to label neurons and axons (Fig. 5A–C). GFP fluorescence was observed in cell bodies and axons (Fig. 5A–C). Within the spinal cord, GFP expression was seen in all laminae from lamina I to X and within the cuneate fasciculus (Fig. 5D and E) indicating labeling of both small and large diameter axons. Continuous labeling of the dorsal roots from the DRG cell bodies extending into the dorsal horn of the cervical spinal cord was also clearly observable (Fig. 5F). Sagittal sections at up to two spinal levels from injected DRGs were also examined and revealed GFP fluorescence in a large number of ascending sensory fibers within the dorsal columns (Fig. 5H). GFP fluorescent labeling was also clearly seen in the sciatic nerve innervating DRGs injected with scAAV2-GFP (Fig. 5I). In summary, DRG neuronal transduction by scAAV2-GFP results in GFP transport into both the peripheral and central projections of sensory neurons from a single ganglion.

### Comparison of Transgene Expression of scAAV2-GFP with ssAAV2-mCherry Vectors in the DRG

**Figure 6:**
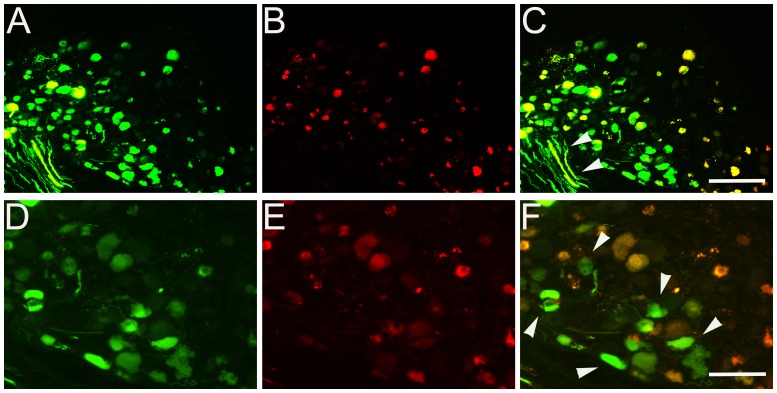
Comparison of transgene expression of scAAV2 with ssAAV2 vectors in DRG. A mixture of scAAV2-GFP and ssAAV2-mCherry vectors was injected into the cervical level DRGs of adult rats. DRG were analyzed one week after viral delivery. GFP (A and D) and mcherry (B and E) expressed simultaneously in prepared DRG samples. Some neurons in DRG and dorsal root axons were only clearly labeled with the scAAV2-GFP vector, and not with the ssAAV2-mCherry vector (arrows in C and F). Scale Bars: 200 µm (A–C), 100 µm (D–F).

To compare self-complementary (sc) and single-stranded (ss) adeno-associated viral vectors for neuronal transduction in the DRG, scAAV2-GFP and ssAAV2-mCherry vectors were prepared to the same titer and equal amounts of scAAV2-GFP and ssAAV2-mCherry viral particles were injected into the same DRGs. Sections of DRG sample were analyzed 1 week after viral delivery and comparison of transgene expression of scAAV2-GFP (Fig. 6A and D) with ssAAV2-mCherry (Fig. 6B and E) was performed on the same sections. Expression of GFP was seen at higher levels in the DRG neurons compared with mCherry expression. Overlaid images showed most DRG neurons were transduced by both vectors (Fig. 6C and F). In high-magnification images, only GFP was expressed independently in some DRG neurons without ssAAV-mCherry expressed (arrowhead) and strong GFP expression, without mCherry expression, was detectable in dorsal root axons (Fig. 6C and F). To control for saturation of GFP in our images, we also injected scAAV2-GFP and ssAAV2-GFP separately into different animals. We found similarly high expression of scAAV2-GFP and low expression of ssAAV2-GFP (data not shown).

### Implication of scAAV2-GFP Vector for Tracing Axonal Regeneration in CST Injury Model

**Figure 7:**
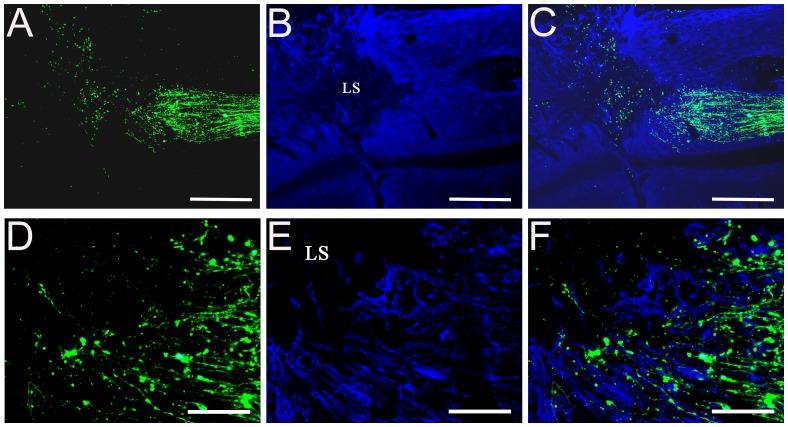
Implication of scAAV2-GFP for labeling CST axons after injury. Dorsal column lesion resulted in a corresponding loss of CST axons, as visualized by anterograde transport of scAAV2-GFP at 4 weeks after viral injection into the sensorimotor cortex (A and C). GFAP staining of the section was to label the lesion site and cavity (B and E). Low magnification images shown in A–B, Corresponding high-magnification images shown in D–F. Scale Bars: 500 µm (A–C), 200 µm (D–F).

To examine whether axonal labeling of scAAV2-GFP could be an appropriate tool for visualizing axonal regeneration after spinal cord injury, we performed a dorsal column lesion to transect fibers of the CST at the cervical level. The dorsal column lesion model has been widely used to investigate axon regeneration [14], [15], [16]. In these animals, scAAV2-GFP was injected into the sensorimotor cortex and GFP expression was examined at the lesion site in sagittal sections. We found that GFP-labeled CST fibers (Fig. 7A and D) labeled all the way to the lesion site, which was localized by GFAP staining (Fig. 7B and E). High magnification images showing the morphology of GFP labeled CST axons in the terminal areas and some axons indicated dieback with swollen terminals apparent at their endpoints (Fig. 7D and F). We did not perform staining to increase the GFP signal. In certain cases, weak GFP expression in individual fibers or higher background may be overcome using a GFP specific antibody.

### scAAV2-GFP Vector for Tracing Axonal Regeneration in a Dorsal Root Crush Model

**Figure 8:**
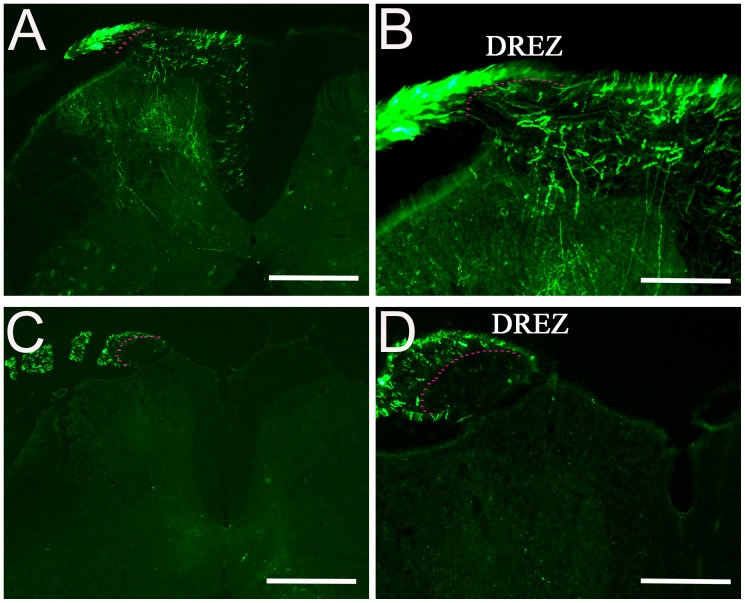
Implications of scAAV2-GFP labeling in dorsal root axons after crush. GFP-labeled axons were examined after dorsal root crush injury. In un-injured animals, GFP-labeled axons enter the spinal cord from the entry zone along with other axons (A and B). After dorsal root crush, cross sections of injured dorsal root with attached spinal cord showing GFP-expressing axons stopped in the DREZ (G and H). DREZ boundary is approximated with a dashed line. Scale Bars: 500 µm (A and C), 200 µm (B and D).

Primary sensory axons injured by a dorsal root crush fail to regenerate into the spinal cord and regenerated axons of dorsal roots stop at the dorsal root entry zone (DREZ), the interface between the CNS and PNS. Many studies have previously examined regeneration of dorsal root axons into the DREZ to identify key molecules involved in axon regeneration [17], [18], [19]. In this experiment, we used this injury model to examine the regeneration of GFP-labeled axons through the DREZ and into the spinal cord after dorsal root crush injury. For these experiments, the dorsal roots were crushed unilaterally at the lumber level and two DRGs (L4, L5) were injected with scAAV2-GFP. In the normal uninjured animal, scAAV2-GFP labeled axons were found in spinal cord laminae I to III transported through the DREZ (Fig. 8A and B, dashed line in B). After a dorsal root crush injury, GFP-expressing axons were found only in the dorsal root, distal to the DREZ and not in the spinal cord (Fig. 8C and D). High-magnification images of a DREZ after crush showed that GFP-labeled axons stopped at the DREZ (Fig. 8D, dashed line).

### Combination of scAAV2-GFP Viral Vector with BDA Tracer for Labeling of Sensory and Motor Axons

**Figure 9:**
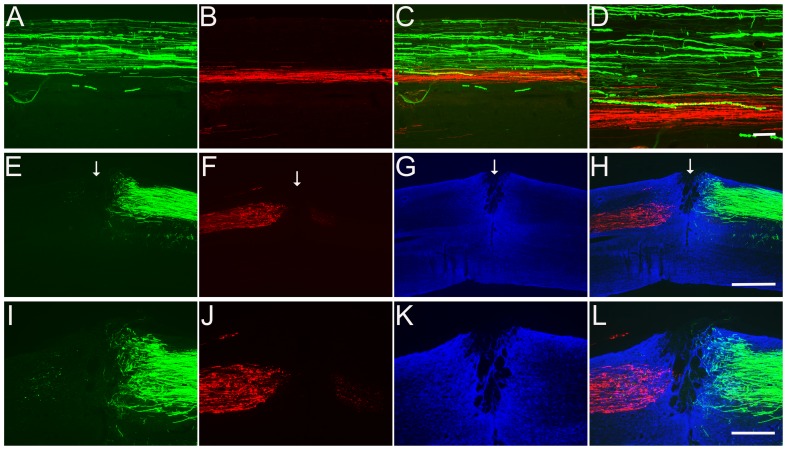
Combining scAAV2-GFP with BDA tracer for labeling sensory and motor axons after injury. Following injections of scAAV2-GFP into the DRG and BDA into the sensorimotor cortex, sections of cervical spinal cord were examined in normal (A–D) and dorsal column lesion animals (E–L). GFAP staining labeling the lesion cavity are shown in blue (G and K). scAAV2-GFP-labeled dorsal sensory axons are shown in green (right of the lesion) (E and I) and BDA labeled CST fibers are shown in red (left of the lesion) (F and J). Scale Bars: 500 µm (A–H), 200 µm (I–L).

In some studies, such as cell transplantation or drug treatments for spinal cord injury, it would be advantageous to use labels that can distinguish between sensory and motor axons. In this study, we combined the viral tracing method with a traditional tracer to separately identify dorsal sensory axons and CST axons in the same sections. We injected scAAV2-GFP into the DRG and BDA into the sensorimotor cortex. Two weeks after BDA or four weeks after scAAV2-GFP injections, cervical spinal cord sections were examined and stained for BDA and GFP labeling. In the normal animal, numerous GFP-labeled dorsal sensory axons and many BDA-labeled CST fibers were found parallel to the longitudinal axis of the spinal cord (Fig. 9. A–D). After dorsal column lesion in the spinal cord, GFP-labeled dorsal sensory axons (right of the lesion) and BDA labeled CST fibers (left of the lesion) reached and stopped at the lesion site, which is indicated by GFAP staining (Fig. 9E–H). High magnification images showed the morphology of GFP labeled sensory axons with BDA labeled CST axons around the lesion areas (Fig. 9I–L).

## Discussion

The AAV vector is one of the most promising viral vectors for human gene therapy. Gene transfer via AAV vectors for the treatment of several CNS disorders, including Parkinson’s disease [20], [21], [22], Alzheimer's disease [23], [24], [25] and lysosomal storage diseases [26] demonstrate the potential of human gene therapy applications. Recombinant Adeno-Associated Virus serotype 2 (AAV2) is the most widely used AAV vector, bringing advantages in long-term gene expression in post-mitotic neurons and a minimal immune response reaction, even after administration of high virus doses [27], [28]. In addition, AAV2 vectors can be packaged with capsid proteins from different AAV serotypes. These alternative serotypes can give unique tropisms and improve transduction efficiency depending on the region of interest and the target neuronal subtype [29], [30], [31]. Taken together, these advantages have led to considerable interest in the AAV2 vector as a gene transfer vehicle in axon regeneration after injury. The conventional AAV2 vector containing the single stranded DNA has to be converted to a double-stranded replicative form, which is a rate limiting step in the replicative cycle of AAV vectors [32], [33]. Double-stranded, self-complementary AAV vectors (scAAV) bypass this step and provide the opportunity to achieve more efficient transduction and faster, more prolonged transgene expression [34], [35]. In this study, a scAAV2 was compared to traditional single-stranded (ss) AAV2 vector for reporter gene expression in the DRG and shown that scAAV2 transgene expression is faster and stronger compared with that achieved by the equivalent ssAAV2 vector. For small tissues in the CNS, such as DRG, a stronger level of expression may be critical, and scAAV vectors can significantly minimize the vector load required to achieve sustained transgene expression. The use of lower viral titers can also reduce the risk of adverse effects due to immune responses caused by the vector itself.

In addition to high transduction efficiency, transgene expression in specific neurons of the nervous system is very important for axonal tracing and gene therapy. To determine whether scAAV2-GFP specifically transduces neurons, we examined a subset of tissues derived from AAV injected animals. These tissues were sectioned and co-labeled with NeuN, a marker for a neuronal nuclear protein or Neurofilament M, a marker for neurons and axons. Our results demonstrate that scAAV2-GFP showed high selectivity for neuronal cells of the sensorimotor cortex, red nucleus and DRG. We did not compare the temporal expression profile or the cellular tropism of different AAV serotype vectors in our present study. In the past few years, several groups have compared the transduction rate and expression level from different AAV serotypes and their results indicated AAV1 (AAV2 vector packaged with serotype 1 capsid proteins) was the optimal serotype for transducing CST neurons and axons projecting through the dorsal CST of the cervical spinal cord [36]. AAV5 has been shown to have the maximum transduction efficiency following direct injection into the dorsal root ganglia, compared to AAV serotypes 1, 2, 3, 4, 6, and 8, and lentivirus (LV) vectors. The time course of GFP expression showed increasing neuronal transduction rates from 12 weeks after AAV5-GFP injections [8]. For RST, quantification of the ratio of transduced RN neurons and RST fibers revealed that AAV1 and AAV8 transduced the highest ratio of RN neurons and the highest number of RST labeling was found in the AAV1-treated animals at 1 month post-injection [37]. Our future studies will utilize the most favorable scAAV serotype to express GFP combined with the appropriate regulatory promoters [38], [39] to achieve the highest-level of sustained GFP expression for axonal tracing.

To assess the ability of AAV to anterogradely transport and label the long-tract axons from the CST, RST and central axons of the DRG, scAAV2-GFP was injected into the sensorimotor cortex, red nucleus and DRGs, respectively. scAAV2-GFP was successfully transported as shown by labeling of GFP-positive fibers in the appropriate descending or ascending tracts extending to the cervical spinal cord. Furthermore, we examined the GFP labeling of axons in two injury models and our data demonstrate that lesioned or regenerated axons could be clearly visualized and easily identified directly under fluorescent microscopy. For visualization of the RST and dorsal root, two weeks provided enough time for infected neurons to express GFP in both their cell bodies and their axons. If the longer axonal tracts such as those of the CST need to be labeled, longer survival times are desired, such as 4 weeks post-injection, as used in the present study. Our observations suggest that scAAV2-GFP is an excellent anterograde tracer for long axonal pathways. In addition to using viral vectors as anterograde labeling, AAV vectors can also retrogradely label axons from their terminals to neuronal bodies. Previous studies indicate that many anterograde tracers also resulted in some retrograde transport. Retrograde infection can occur through active transport of the viral genome from synaptic terminals in the area of injection [40]. In our unpublished data, injection of scAAV2-GFP into the L2 spinal cord retrogradely labeled the RST and their rubrospinal neurons in the brain stem (data not shown). Thus, in the future, the injections of double or triple fluorescence expressing scAAV vectors, such as GFP (green), mCherry (red) and Tomato (red-orange) can be used to provide multiple tracts tracings anterogradely and retrogradely at the same time.

AAV2-mediated gene transfer approaches have been designed for the treatment of spinal cord injury and neurological disorders [16], [20], [21]. Concurrent injection of different AAV2 vectors can co-express therapeutic genes and fluorescent markers like GFP. Because the neuronal cell body of the labeled axons also expresses the therapeutic gene, the resulting regeneration of anterogradely labeled axons will directly demonstrate that this regenerative effect comes from the therapeutic gene [4]. Other studies have shown that GFP labeled axons can be used for the visualization and quantification of sprouting and regeneration of CST axons without tracers. For example, coinjection of AAV8-GFP and AAV8-KLF7 into the sensorimotor cortex was shown to promote CST axonal sprouting and growth [14]. Further, scAAV tract tracing techniques combined with viral-mediated expression of axonal growth promoting genes, such as growth factors [41], [42], [43], mTOR activators [44] or channelrhodopsins (ChRs) [45], [46] will allow axon regeneration to be detected more easily and precisely.

In summary, we report that the development of a recombinant scAAV2 vector carrying a GFP reporter gene can efficiently transduce neurons in the sensorimotor cortex, red nucleus and DRGs, and intensely label their axonal fibers. Following lesions in the dorsal column or dorsal roots, injection of scAAV2-GFP into the sensorimotor cortex or DRGs allows direct visualization of transected or regenerating axons in the lesion site or dorsal root entry zone. The scAAV2-GFP axon tracing technique can also be combined with scAAV2-mediated expression of other genes to directly and precisely assess the transgene effect on axon regeneration.
